# There are three major *Neisseria gonorrhoeae* β-lactamase plasmid variants which are associated with specific lineages and carry distinct TEM alleles

**DOI:** 10.1099/mgen.0.001057

**Published:** 2023-07-12

**Authors:** Tabea A. Elsener, Keith A. Jolley, Eduard Sanders, Martin C. J. Maiden, Ana Cehovin, Christoph M. Tang

**Affiliations:** ^1^​ Sir William Dunn School of Pathology University of Oxford, Oxford, UK; ^2^​ Department of Biology, University of Oxford, Oxford, UK; ^3^​ Arum Institute, Johannesburg, South Africa, and KEMRI-Wellcome Trust Research Programme, Kilfi, Kenya

**Keywords:** *Neisseria gonorrhoeae*, p*bla*, β-lactamase, TEM-1, TEM-135, plasmid typing, Ng_p*bla*ST

## Abstract

*
Neisseria gonorrhoeae* is a significant threat to global health with an estimated incidence of over 80 million cases each year and high levels of antimicrobial resistance. The gonococcal β-lactamase plasmid, p*bla*, carries the TEM β-lactamase, which requires only one or two amino acid changes to become an extended-spectrum β-lactamase (ESBL); this would render last resort treatments for gonorrhoea ineffective. Although p*bla* is not mobile, it can be transferred by the conjugative plasmid, pConj, found in *N. gonorrhoeae *. Seven variants of p*bla* have been described previously, but little is known about their frequency or distribution in the gonococcal population. We characterised sequences of p*bla* variants and devised a typing scheme, Ng_p*bla*ST that allows their identification from whole genome short-read sequences. We implemented Ng_p*bla*ST to assess the distribution of p*bla* variants in 15 532 gonococcal isolates. This demonstrated that only three p*bla* variants commonly circulate in gonococci, which together account for >99 % of sequences. The p*bla* variants carry different TEM alleles and are prevalent in distinct gonococcal lineages. Analysis of 2758 p*bla*-containing isolates revealed the co-occurrence of p*bla* with certain pConj types, indicating co-operativity between p*bla* and pConj variants in the spread of plasmid-mediated AMR in *N. gonorrhoeae *. Understanding the variation and distribution of p*bla* is essential for monitoring and predicting the spread of plasmid-mediated β-lactam resistance in *N. gonorrhoeae *.

## Data Summary

WGS of isolates in this study are deposited in PubMLST with their IDs listed in Table S2, available in the online version of this article.

**Impact Statement**

Plasmid-mediated AMR contributed to the discontinuation of penicillin and tetracycline for treating gonococcal infection. This article is, to our knowledge, the most extensive analysis of the occurrence and distribution of variants of the β-lactamase plasmid, p*bla*, in the WHO priority pathogen *N. gonorrhoeae *, and their carriage of TEM alleles. The TEM β-lactamase is only a few changes away from becoming an ESBL, which would render last resort treatments for gonorrhoea ineffective. Previously, identification of p*bla* variants relied on PCR or restriction digestion patterns which hinders the analysis of large numbers of isolates, while p*bla* fails to assemble from short-read, whole genome sequences due to repeat sequences on the plasmid. We report the development of the p*bla*-typing scheme, Ng_p*bla*ST, to identify variants from short-read sequence data. Ng_p*bla*ST is now available on the PubMLST Neisseria database (https://pubmlst.org/neisseria/) and can be used to unambiguously identify the plasmid and its variants in gonococcal isolates.

## Introduction

The sexually transmitted infection (STI) gonorrhoea is a major public health concern, with a yearly global incidence of over 80 million cases [1]. Untreated, *Neisseria gonorrhoeae* can cause serious complications such as disseminated infection, pelvic inflammatory disease, and infertility. Without an available vaccine, the management of gonococcal disease depends on effective antimicrobial treatment. However, strains of *N. gonorrhoeae* have developed resistance against all first- or second-line antibiotics [2]. Currently, monotherapy with the β-lactam ceftriaxone is the recommended treatment for gonococcal infection in the UK and USA [3, 4]. Resistance to β-lactam antibiotics in the gonococcus can be mediated by chromosomal genes such as mosaic *penA* alleles [5], or plasmid-encoded TEM β-lactamase. Whilst chromosomal resistance to β-lactams is more frequent in high-income countries (HICs), there is a high prevalence of plasmid-mediated resistance in low and middle-income countries (LMICs) [6].

The gonococcal β-lactamase plasmid (p*bla*) encodes TEM β-lactamases, which confer resistance to penicillin, ampicillin and cephaloridine [7, 8]. TEM-1 is the most frequently found β-lactamase in gonococci [6] and requires only two amino acid substitutions to become an extended spectrum β-lactamase (ESBL) [9]; one of these substitutions is already present in TEM-135 [10]. The emergence and spread of a transmissible ESBL is a significant public health threat, as it would render current the recommended treatment ineffective and undermine our ability to cure and control gonococcal infection.

p*bla* is a 3.2–9.3 kb plasmid, which carries its own origin of transfer (*oriT*), MobA relaxase and MobC [11], allowing its transfer by the gonococcal conjugative plasmid, pConj [12]. p*bla* carries three distinct origins of replication, *ori1*, *ori2* and *ori3*, and two replication initiation proteins RepA and RepB, which facilitate replication from *ori1* and *ori2*/*ori3*, respectively [13], allowing its replication in *N. gonorrhoeae *, *Escherichia coli *, *Salmonella enterica* serotype Minnesota, and *Haemophilus influenzae *. Seven variants of p*bla* have been described [14–16] and are thought to have emerged from the prototypical 7.4 kb plasmid usually referred to as the Asia variant (p*bla*.As). The variants differ in the presence of replication and mobilisation genes [14] and could vary in their capacity to be transferred. Additionally, it has been reported that some variants (e.g. p*bla*.Rio) are primarily associated with TEM-135 [16, 17].

Previously, p*bla* variants have been identified by their molecular weight, restriction digestion pattern [18, 19] or PCR [20, 21] which require DNA from isolates, hindering large-scale analysis. Therefore, the spread of p*bla* variants in the gonococcal population, and their association with TEM alleles and pConj variants are unknown. Furthermore, specific PCR assays developed to distinguish between the three earliest variants, do not identify later variants [16], and have led to discrepancies between sequencing data and PCR typing results even for WHO reference strains [22]. In this work, we established and implemented a typing scheme for p*bla* based on the characteristic gene presence/absence pattern of variants and propose a numeric nomenclature for p*bla* variants, avoiding the use of geographic locations as recommended by the WHO [23]. The typing scheme, Ng_p*bla*ST, is publicly available on the PubMLST [24] *Neisseria* database (https://pubmlst.org/neisseria/) and can be used to identify the presence of plasmid variants in gonococcal isolates. We analysed the distribution of p*bla* in 15 532 isolates and found that only three of the seven previously described p*bla* variants circulate in gonococci at any appreciable frequency. Furthermore, we show that p*bla* variants are associated with TEM alleles, pConj types, and particular gonococcal lineages.

## Methods

### Whole genome sequence data

Gonococcal whole genome sequence (WGS) data in the PubMLST database (*n*=15 664, accessed 28 July 2022) were analysed on the BIGSdb platform [24], discarding WGS from isolates with ambiguous ribosomal multilocus sequence type (rMLST, e.g. incorrect assignment of species) [25] and removing duplicated entries. This resulted in a dataset of 15 532 isolates from 66 countries, including all six WHO regions, and spanning the years 1928 to 2022 (Table S1 and 2, Fig. S1).

### Plasmid characterisation

pJD4 (NC_002098.1; Asia p*bla* variant) [26] was analysed as a representative p*bla*. The plasmid sequence was blasted (BLASTn with expect threshold: 0.05, word size: 28, mach/mismatch scores: 1,–2, linear gap costs) against the NCBI nucleotide collection [27] to assess the host range of p*bla*. Individual open reading fram (ORF) amino acid sequences were blasted (BLASTp with expect threshold: 0.05, word size: 6, BLOSUM62 scoring matrix, gap costs: 11 existence, 1 extension) against the NCBI non-redundant protein sequence database [27]. Furthermore, p*bla* RepA and RepB amino acid sequences were compared to RepA of the IncFII plasmid R1 [28] and IncW plasmid R388 [29] using ClustalW with default parameters [30]. Plasmid Mob type was identified with MOB-typer version 3.1.0 [31, 32]. For the characterisation of NEIS2964, HHpred [33, 34] was used for homology detection and structure prediction with default parameters (HHblits=>UniRef30, three MSA generation iterations, e-value cutoff: 1e-3, MAC realignment threshold: 0.3). Hydrophobicity plots were generated with ProtScale [35] using the scale Hphob./Kyte and Doolittle. The conserved neighbourhood of NEIS2964 was assessed using WebFlaGs [36] with BLASTp against NCBI RefSeq database (updated 29 January 2021, e value 1e-10) and three jackhmmr iterations. Initial hits of conserved neighbours (WP_140450222.1, WP_010904468.1 and WP_012881329.1) were investigated as above to assess their co-occurrence with NEIS2964.

### Analysis and typing of variants

Representative sequences of p*bla* deletion variants (Genbank accession numbers NZ_LT591905.1, DQ355980.1, HM756641.1, KJ842484) were mapped onto pJD4 (NC_002098.1) in Snapgene v6.1.1 (Insightful Science; available from snapgene.com) to identify variant-specific deletions and establish the gene presence/absence matrix for the typing scheme. The published insertion/repeat sequences U20421 and U20422.1 were blasted (BLASTn, word size: 11, match/mismatch scores: 2,–3, linear gap costs) against the isolate dataset (Table S2) in the PubMLST database [24]. Hits with an alignment length of >500 bp (IS*5* sequence U20421) or >50 % (repeat sequence U20422.1) were assessed for their genomic context by extracting the respective contig from the sequencing bin and identifying the co-located ORFs. Furthermore, the IS*5* sequence was blasted (BLASTn, word size: 28, match/mismatch scores: 1,–2, linear gap costs) against the NCBI nucleotide collection [27].

p*bla*-carrying gonococci (*n*=2758, accessed 28 July 2022) were identified by the presence of NEIS2960, which encodes a hypothetical protein and is found in all plasmid variants [6]. Gene absence was confirmed by blasting available alleles against plasmid sequences using the sequence tag scan function on PubMLST with default parameters (minimum identity: 60 %, minimum alignment length: 50 % and BLASTn word size: 20). Plasmids were typed according to the characteristic gene presence/absence pattern of variants with incompletely assembled loci considered as present.

To check the accuracy of the typing scheme, we used p*bla* short-read sequences of p*bla*-containing WHO reference strains (WHO M, WHO N, WHO O), previously characterised by long-read sequencing [37]. We confirmed the p*bla* types in these strains by PCR using previously described primers [20] (0.25 µM) and Herculase II Fusion DNA Polymerase (Agilent) with the following cycling conditions: 92 °C for 2 min, followed by 30 cycles of 92 °C for 20 s, primer annealing at 50 °C for 20 s, elongation for 3 min at 68 °C, and a final extension at 68 °C for 8 min. Additionally, 16 gonococcal isolates, from nine Ng_cgc_400 lineages, eight countries and isolated from 1986 to 2015, containing different p*bla* variants were randomly selected from the gonococcal dataset (Tables S2 and S3) and typed using the scheme. p*bla*-containing contigs were then extracted from the sequencing bin and aligned to pJD4 (NC_002098.1) to define deleted regions and confirm the completeness of the remaining sequences. Sequencing quality was assessed by comparing sequencing parameters, such as number of contigs, N50, L50, percentage of alleles designated, percentage of loci tagged of isolates, which are accessible on BIGSdb.

For further analyses of the distribution of TEM alleles amongst p*bla* variants, typable plasmids with incomplete loci were removed from the analysis, resulting in a dataset of 2157 p*bla* sequences.

### Analysis of the distribution of p*bla* variants

*N. gonorrhoeae* population structure was resolved using core genome MLST (cg-MLST) [38]. This scheme groups gonococcal isolates according to allelic designations in 1668 loci core to the gonococcus. Each different allele at every locus is given a unique integer and isolates are grouped into single-linkage clusters according to the number of allelic differences. A cut-off of 400 allelic differences between individual isolates resolves the population into discrete *N. gonorrhoeae* core genome clusters (Ng_cgc_400) which are stable over time [38]. A minimal spanning tree was generated with GrapeTree [39], clustering isolates according to core genome allelic differences. NEIS2220 (*trbM*) was used to detect the presence of pConj in individual isolates [6]. pConj types were defined based on the pConj typing scheme with a cut-off of five allelic differences (Ng_cp_5) [6]. Where the Ng_cp_5 could not be assigned, plasmids were clustered based on pConj core genome loci [6] using GrapeTree [39], and pConj types were identified manually.

### Statistical analysis

Data manipulation and analysis were performed in R version 4.1.1 using base R and the tidyverse package [40]. To calculate odds ratios (OR), the odds.ratio function from the epitools package [41] was used. Plots were generated with ggplot2 [42]. A *P* value of <0.01 was considered statistically significant.

## Results

### Characterisation of gonococcal p*bla*

**Fig. 1.**
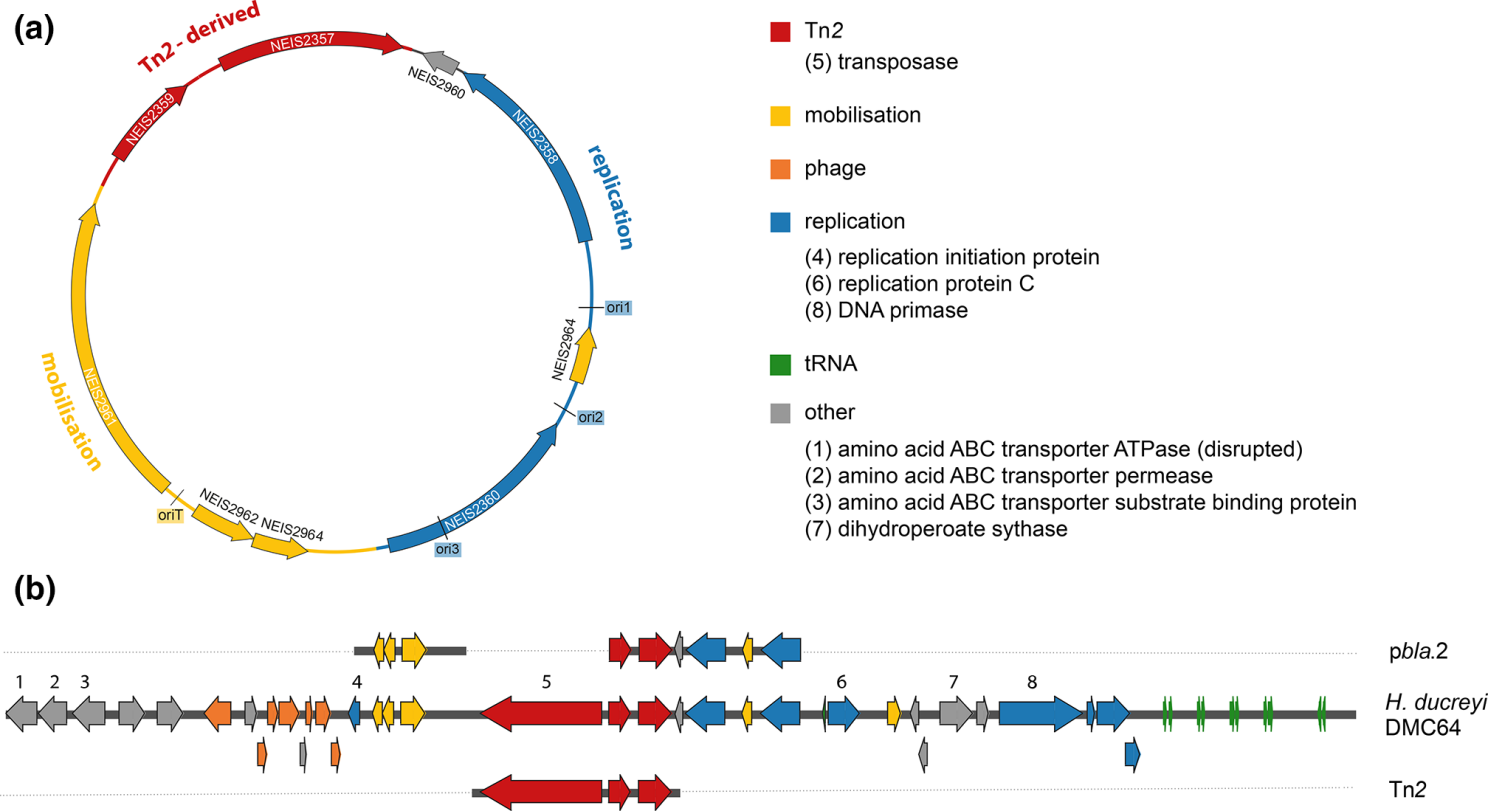
(**a**) Gene organisation of p*bla*.2 (p*bla*.As). ORFs are indicated as arrows with colours reflecting gene products; grey, hypothetical proteins; yellow, mobilisation proteins; red, Tn*2*-derived genes including *bla*TEM (NEIS2357); blue, replication initiation proteins. (**b**) p*bla*.2 sequences can be found in the *H. ducreyi* chromosome. Genomic organisation of p*bla* in *H. ducreyi* DMC64 (Genbank accession number CP11230) is shown. The aligned p*bla*.2 sequence (Genbank accession number NZ_LT591911) is indicated above and Tn*2* (Genbank accession number HM749967.1) shown below. Tn*2* is intact in *H. ducreyi *, while a truncated version is present in gonococcal p*bla*.

Seven variants of p*bla* have been described in the gonococcus [14, 16, 43], ranging in size from 3.2 to 9.3 kb due to duplication and deletion events. The prototypical 7.4 kb plasmid, often referred to as the Asia variant [14], contains nine loci that can be functionally grouped into Tn*2*-derived, replication, and mobilisation regions (Fig. 1a). The plasmid contains 34 % of Tn*2* [44], including *bla*_TEM_, 84 % of the *tnpR* resolvase gene, and the right inverted repeat [45, 46].

The replication region of p*bla* contains NEIS2358 (*repA*) and NEIS2360 (*repB*) encoding replication initiation proteins (Rep) that are required for initiation of replication at *ori1* and *ori2/ori3*, respectively [13]. The three origins belong to the iteron family [13], with approximately half of the sequence consisting of an array of ~20 bp repeats. These serve as binding sites for the Rep proteins [47]. p*bla* has been classified as an IncW plasmid with a silent IncFII replicon which is used when the *ori2/ori3* region is absent [13]. However, RepA shares less than 17 % amino acid identity with RepA from the characterised IncFII plasmid R1 [28], and RepB has only 17.9 % amino acid identity with the IncW plasmid R388 RepA [29]. MOB-typer [31, 32], which probes a collection of plasmid-derived replication protein sequences against the query sequence, instead classifies p*bla* as an IncP plasmid.

The mobilisation region of p*bla* contains an origin of transfer (*oriT*), flanked by genes encoding a relaxase (MobA, NEIS2961) and MobC (NEIS2962) [11]. Mob typing characterises plasmids according to their N-terminal relaxase domain [48], and identified p*bla* as a MobQ family plasmid. MobQ plasmids have been reported to be efficiently mobilised by helper plasmids belonging to a variety of Inc groups, leading to their spread across different phyla [48]. Therefore, we investigated the host range of p*bla* and found it is largely limited to *N. gonorrhoeae *, with only two clinical *Neisseria meningitidis* isolates possessing the plasmid [49]. Interestingly, we identified p*bla* sequences in the chromosome of seven of the 30 available *Haemophilus ducreyi* sequences in the NCBI nucleotide database. *H. ducreyi* strains DMC64 (Genbank accession: CP011230.1) and 33 921 (Genbank accession: CP011228.1) [50] are annotated with intact p*bla* in their chromosome adjacent to phage-related genes (Fig. 1b). Unlike in p*bla*, Tn*2* is intact in the *H. ducreyi* sequences and includes the transposase gene which is absent from gonococcal p*bla*. Furthermore, p*bla* GC-content (38.3 %) is similar to *H. ducreyi* chromosomal DNA (37.9–38.6 %) [50], whilst the *N. gonorrhoeae* chromosome GC-content is 52.5 % [51].

NEIS2964 is present in two copies on the 7.4 kb p*bla* and is predicted to encode an 86 amino acid protein of unknown function. Hydrophobicity plots of NEIS2964 indicate it has two domains; an N-terminal transmembrane segment and a charged C-terminal domain (Fig. S2). Neighbourhood analysis revealed a conserved localisation of NEIS2964 with *mob* and *rep* (Fig. S3). However, whilst *repB* is frequently found without NEIS2964-related genes, *mobA* and *mobC* show a consistent association with NEIS2964 (Fig. S3). This suggests a role for NEIS2964 in plasmid mobilisation.

NEIS2960 is present in all described p*bla* variants [6] and shows high sequence conservation (>98.2 % nucleotide identity) amongst plasmid sequences (*n*=2758), with 97.7 % of plasmids carrying NEIS2960 allele 1. Sequence analysis reveals NEIS2960 is a predicted antitoxin belonging to the VbhA family (Genbank accession: WP_164823310.1).

### p*bla* typing reveals that three major variants circulate in gonococci

Understanding the variation and prevalence of p*bla* is essential for monitoring and predicting the spread of plasmid-mediated β-lactam resistance in the gonococcus. However, repeat sequences on the plasmid hinder p*bla* assembly from short-read sequencing data. Therefore, we developed a typing scheme (Ng_p*bla*ST; available on https://pubmlst.org/neisseria/) based on the characteristic gene presence/absence pattern of p*bla* variants, which does not require plasmid assembly (Table 1). We propose a new p*bla* nomenclature which avoids the use of geographic regions, as this can convey misleading information about origin and could be stigmatising [52]. This implements the recent WHO recommendations on renaming infectious diseases [23] and number the variants in the order of when they were first reported (Table 1). Therefore, p*bla*.1 corresponds to p*bla*.Af, p*bla*.2 to p*bla*.As, p*bla*.3 to p*bla*.Rio, p*bla*.4 to p*bla*.Jo and p*bla*.5 to p*bla*.Au.

**Table 1.** Gonococcal p*bla* variants with their defining features

	Previous name	First report	Size (kb)	Molecular wt (MDa)	Defining gene absence pattern	Reference
p*bla*.1	p*bla*.Af	1976	5.6	3.2	*repB*	[71]
p*bla*.2	p*bla*.As	1976	7.4	4.2–4.4		[72]
p*bla*.3	p*bla*.Rio	1984	5.1	2.9–3.05	*mobA*, *mobC*	[73]
p*bla*.4	p*bla*.Jo	2011	5.4	na	*repB*, *mobC*, NEIS2964	[43]
p*bla*.5	p*bla*.Au	2012	3.2	na	*repA*, *mobA*, *mobC*, NEIS2964	[16]

**Fig. 2.**
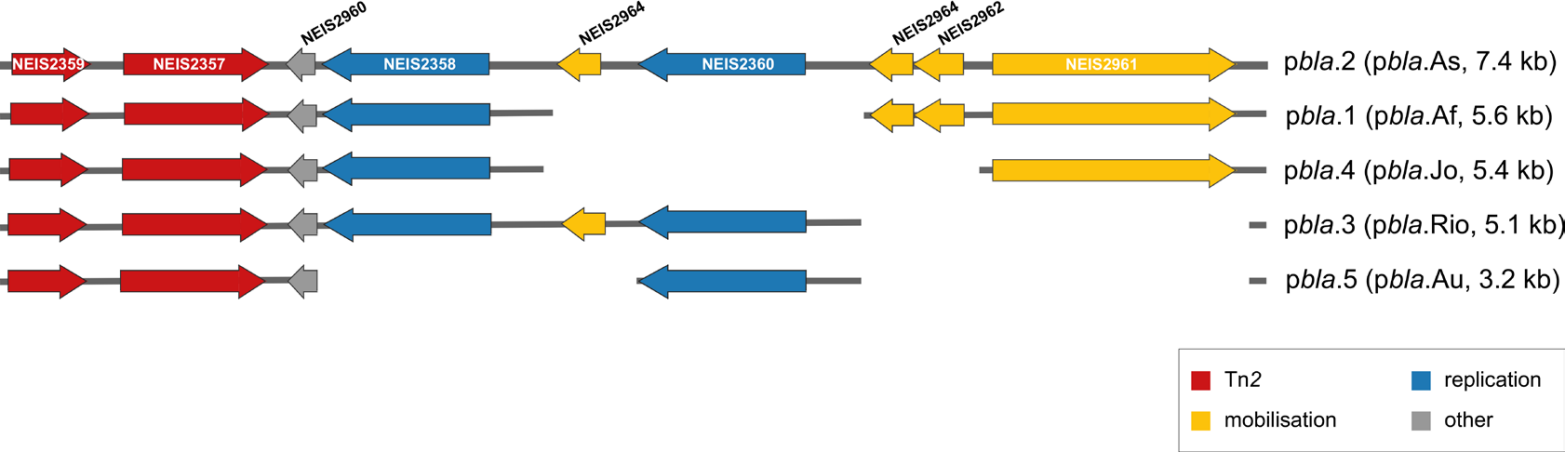
Schematic representation of the p*bla* deletion variants aligned to p*bla*.2 (p*bla*.As). Grey line indicates aligned regions with arrows representing ORFs coloured according to their gene products; grey, hypothetical proteins; yellow, mobilisation proteins; red, Tn*2*-derived genes including *bla*TEM (NEIS2357); blue, replication initiation proteins.

We first analysed published sequences of p*bla* variants and identified their differences based on the presence/absence of genes in the 7.4 kb p*bla*.2 (previously described as the Asia variant) [26]. Two insertion variants and four deletion variants of p*bla*.2 have been described. The insertion variants [18, 19], previously referred to as New Zealand and Nîmes variants, have a duplication or IS*5* insertion downstream of *bla*TEM, respectively. Both have only been reported on a single occasion [18, 19]. We analysed available gonococcal and p*bla* sequences (*n*=15 532) and the p*bla* sequences in 2758 of these isolates (Table S1) for the presence of the duplicated sequence and IS*5*. We obtained a single match for the duplicated sequence in p*bla*-containing gonococcal isolates. However, closer investigation revealed that this isolate (GHA-TMH-537) shows an uncharacteristic duplication of NEIS2960, NEIS2357, NEIS2359 and NEIS2360 and a deletion of NEIS2961 and NEIS2962. Additionally, IS*5* is not present in *Neisseria* spp. or in *Haemophilus* spp., but is found on the chromosome of the *E. coli* strain from which the insertion variant was isolated following transformation with gonococcal plasmid DNA [19]. Therefore, p*bla*-insertion variants are rare and may be experimental artefacts [19].

p*bla*.1 (5.6 kb, previously the African variant) [37] and p*bla*.4 (5.4 kb, previously the Johannesburg variant) [43] harbour characteristic deletions in the replication region. p*bla*.1 is structurally identical to p*bla*.2 except for the loss of a 1 826 bp fragment (Fig. 2, Table 1), which spans NEIS2960 (*repB*), NEIS2964, *ori2* and *ori3*. p*bla*.4 has a 2 560 bp deletion that includes *ori2* and *ori3*. In addition to genes absent from p*bla*.1, p*bla*.4 lacks NEIS2962 (*mobC*) and the second copy of NEIS2964 (Fig. 2, Table 1).

p*bla*.3 (5.1 kb, previously the Rio/Toronto variant) [53] and p*bla*.5 (3.2 kb, previously the Australia variant) [16] have a 2 270 bp deletion spanning the *mob/oriT* region (Fig. 2, Table 1). p*bla*.5 is the smallest p*bla* variant reported to date and has lost a further 1 885 bp comprising *ori1* and NEIS2358 (*repA*), as well as NEIS2962 and NEIS2964.

To check the accuracy of Ng_p*bla*ST, we examined available whole genome, short-read sequences of WHO reference strains which are known to harbour p*bla* and have been characterised by long-read sequencing [37]. Ng_p*bla*ST correctly identified the p*bla* variant in every instance from the short-read sequences alone. Additionally, we analysed short-read sequences from 16 gonococcal isolates containing p*bla* (Table S2) by extracting plasmid-loci containing contigs from the sequencing bin and mapping them against the p*bla*.2 reference sequence; this confirmed the exact variant-specific deletions in all tested variants (not shown).

With Ng_p*bla*ST, we analysed the 2758 p*bla*-carrying isolates in our dataset which originate from 50 countries representing all six WHO regions and were recovered between 1979 and 2022 (Table S1). Although it is possible to distinguish between p*bla* variants based on only four genes (NEIS2961, NEIS2962, NEIS2964 and NEIS2360), all plasmid loci were included in our scheme to avoid incorrect allocation of variants due to low sequencing quality. The p*bla* variant was assigned in 74.6 % of isolates. The remaining sequences showed uncharacteristic gene presence/absence patterns due to poor sequencing quality. The sequences of isolates with uncharacterised p*bla* had a significantly lower N50 than isolates with typed p*bla* (average N50 untyped=59 578.6, average N50 typed=78 270.6, two sample t-test *P*<0.001). When assembling genomes from the longest to the shortest contig, the N50 is the length of the shortest contig when half the total genome has been assembled [54]. Of the typed isolates (2 054), only nine and three isolates carry p*bla*.4 and p*bla*.5, respectively (Table S2), while 1433 (69.7 %) carry p*bla*.1, 326 (15.8 %) p*bla*.3 and 286 (13.9 %) p*bla*.2.

While p*bla*.1 is present globally and across the gonococcal population, other p*bla* variants show distinct geographic patterns. p*bla*.2 is associated with China (OR=32.0, *P*<0.001), while p*bla*.3 is more frequently found in HICs (OR=3.0, *P*<0.001) [33].

### p*bla* variants carry distinct TEM alleles

**Fig. 3.**
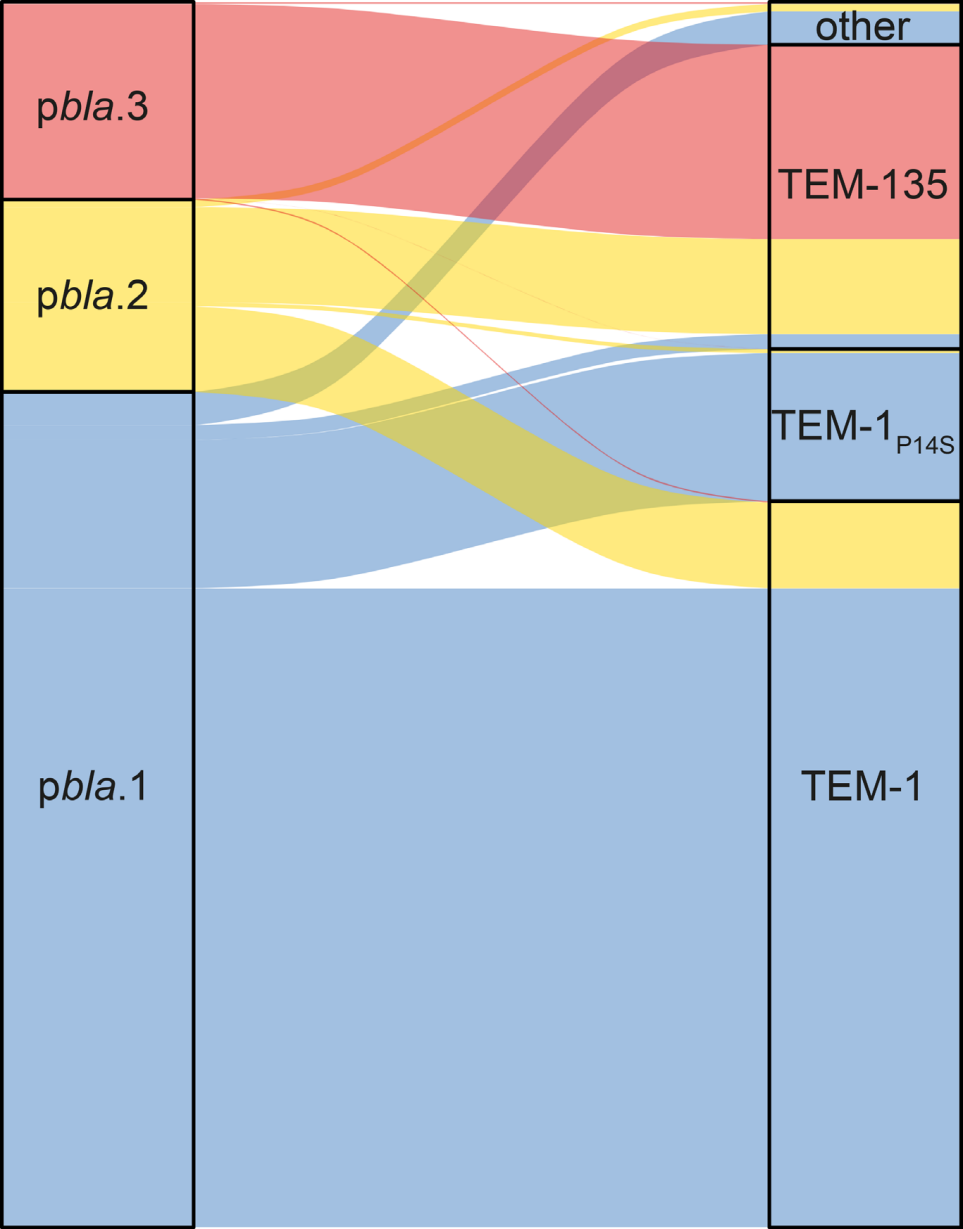
Sankey diagram displaying the proportion of p*bla* variants relative to each other (left) and linking them to their associated TEM alleles (right). p*bla*.3 is significantly associated with TEM-135. p*bla*.2 carries TEM-1 and TEM-135 at equal frequencies, whilst p*bla*.1 mainly carries TEM-1 and TEM-1 with a P14S substitution.

All p*bla* variants carry *bla*TEM, conferring resistance to penicillins. There are 59 *bla*TEM alleles in the 2157 p*bla* with complete *bla*TEM sequences in the PubMLST database. TEM-1 (encoded by allele 3) is the most prevalent, accounting for 59.6 % (1286/2157) of sequences, followed by TEM-135 (allele 2; 24.3 %, 525/2157) and allele 6 (11.1 %, 240/2157). TEM-135 has an M182T substitution, which increases the stability of the enzyme and often precedes further mutations extending the range of substrates [55, 56]. Individual isolates carry alleles with substitutions in addition to M182T at positions P14, R61, L201, A224 and A248. These substitutions have not been characterised or are not known to confer increased resistance to β-lactams. Allele six has a P14S substitution in its leader sequence and is found in a subpopulation of p*bla*.1.

p*bla*.3 is significantly associated with TEM-135 (found in 98.3 % of p*bla*.3; OR=66.9, *P*<0.001) (Fig. 3). p*bla*.2 carries TEM-1 and TEM-135 at an approximately equal frequency (44.5 and 49.5 %, respectively), whilst p*bla*.1 mainly carries TEM-1 (76.5 % of isolates with this p*bla*), TEM-1_P14S_ (17.7 %) and rarely TEM-135 (1.78 %). Our results demonstrate that TEM-1, TEM-135 and TEM-1_P14S_ are the major TEM variants in gonococci, and p*bla* variants differ in their TEM allele carriage (Fig. 3).

### p*bla* variants are associated with specific gonococcal lineages

We next analysed the distribution of p*bla* variants amongst 15 532 *N *. *gonorrhoeae* isolates originating from 66 countries and isolated from 1928 to 2022 (Table S1, Fig. S1). p*bla* is present in 17.8 % of isolates (2 758/15 532), with significant differences in prevalence between geographic regions (chi^2^-test, *P*<0.001, Table 2). Significantly high rates of p*bla*-carriage were found in isolates from Africa (58.4 %, 185/317; OR=6.9, *P*<0.001). To define the distribution of p*bla* amongst gonococcal lineages, we grouped isolates into *N. gonorrhoeae* core genome clusters (Ng_cgc_400) [38]. Strikingly, isolates from Ng_cgc_400 21 and 34, which are prevalent in Africa but also found in Asia, Europe, and North America, show a high p*bla* prevalence (>50 %) irrespective of their origin, indicating p*bla* carriage is a feature of a lineage, rather than the region from where a strain had been isolated.

**Table 2.** p*bla* prevalence varies significantly between geographic regions. The percentage of isolates carrying p*bla* is indicated as well as the prevalence of different plasmid variants. Absolute numbers are shown in brackets. p*bla*.1 is the most prevalent variant in all regions except for Asia, where p*bla*.2 dominates. p*bla*.3 is more frequently found in Europe and North America

	p*bla* prevalence (%)		p*bla* variant (%)		
		p*bla*.1	p*bla*.2	p*bla*.3	Other/undefined
Africa	**58.4** (185/317)	85.4 (158)	5.9 (11)	3.8 (7)	4.9 (9)
Asia	24.2 (334/1383)	37.7 (126)	47.0 (157)	86.6 (22)	8.7 (29)
Europe	20.0 (1780/8879)	47.9 (853)	4.9 (87)	13.4 (239)	33.8 (601)
North America	7.9 (265/3337)	65.7 (174)	5.3 (14)	18.1 (48)	10.9 (29)
South America	22.3 (110/493)	67.3 (74)	0.9 (1)	3.6 (4)	28.2 (31)
Oceania	6.4 (65/1011)	61.5 (40)	15.4 (10)	4.6 (3)	18.5 (12)

p*bla*.3 is predominantly found in Ng_cgc_400 25, 298 and 391, which cluster together on a minimum spanning tree (Fig. 4a, Table 3). p*bla*.2 is mainly present in Ng_cgc_400 122 and 29 (Fig. 4b, Table 3). While p*bla*.1 is found across the gonococcal population, TEM-1_P14S_ carrying p*bla*.1 is confined to Ng_cgc_400 33 (Fig. 4c, Table 3).

**Table 3.** Odds ratios of p*bla* variants in distinct gonococcal core genome clusters. The odds ratios were calculated as the odds of p*bla*-carrying isolates in the core genome clusters (cgc) to carry the indicated variant divided by the odds of any p*bla*-carrying isolate to harbour the specific variant, and the *P*-value is given in brackets. The 95 % confidence intervals (lower, upper in brackets) and the *P* values are given

p*bla* variant	Lineage		
	Ng_cgc_400 33	Ng_cgc_400 29, 122	Ng_cgc_400 25, 298, 391
p*bla*.1 TEM-1_P14S_	509.8 (267.0, 1083.7) *P*<0.001	0.2 (0.2, 0.3) *P*<0.001	0.004 (0.001, 0.01) *P*<0.001
p*bla*.2	0.02 (0.001, 0.1) *P*<0.001	14.0 (9.7, 19.2) *P*<0.001	0.1 (0.1, 0.3) *P*<0.001
p*bla*.3	0	0.1 (0.002, 0.03) *P*<0.001	92.25 (64.8, 133.3) *P*<0.001

**Fig. 4.**
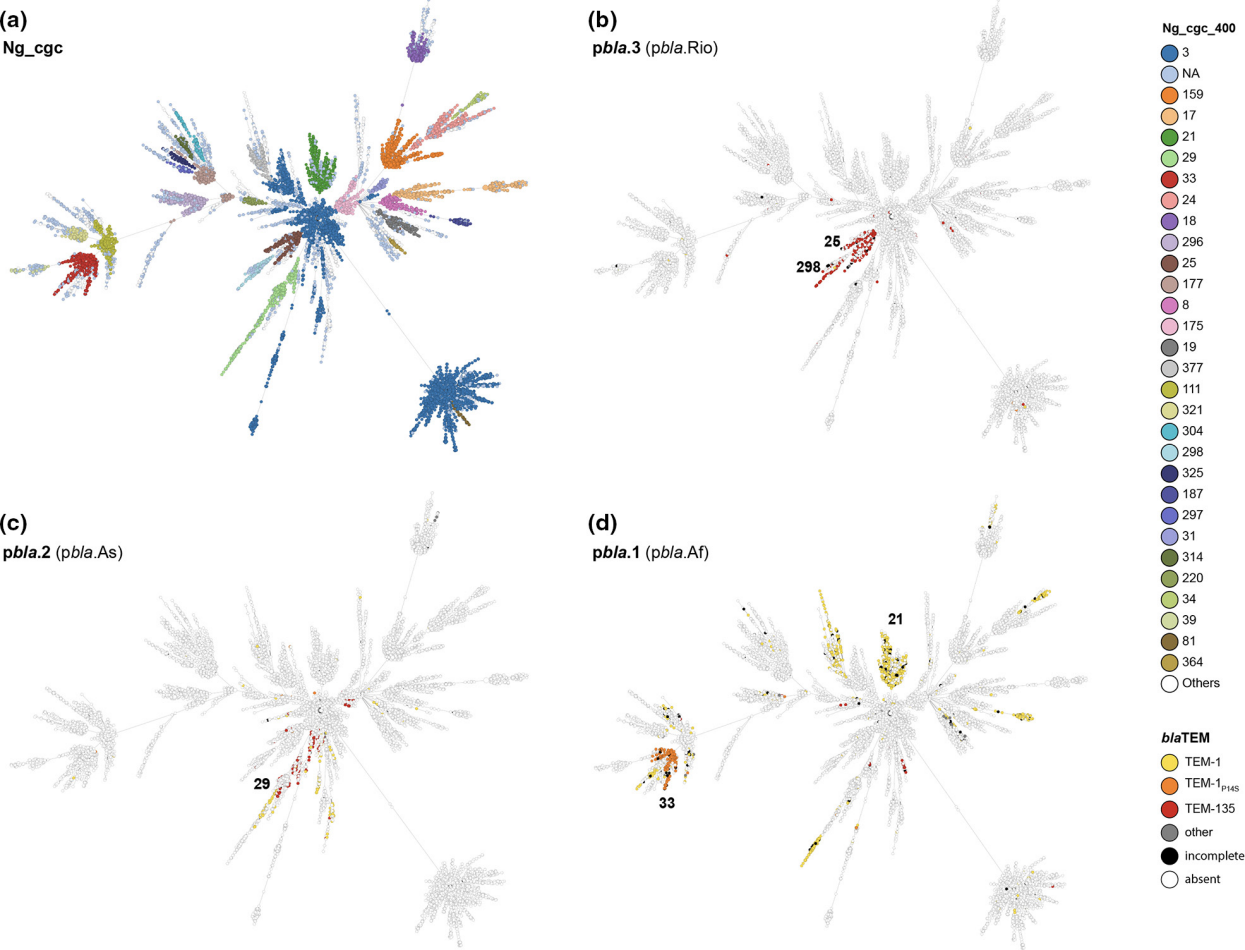
Minimal spanning trees of 15 532 gonococcal isolates based on core genomic allelic differences showing the association between p*bla* variants, TEM alleles and gonococcal lineages (**a**). Individual trees show the distribution of p*bla*.1 (p*bla*.Af) (**b**), p*bla*.2 (pb*la*.As) (**c**) and (**d**) p*bla*.3 (p*bla*.Rio) in gonococci with characteristic Ng_cgc_400 indicated. Circles represent single isolates that are coloured according to Ng_cgc_400 (**a**) or TEM allele carried (**b-d**).

### p*bla* variants co-occur with distinct pConj variants

**Fig. 5.**
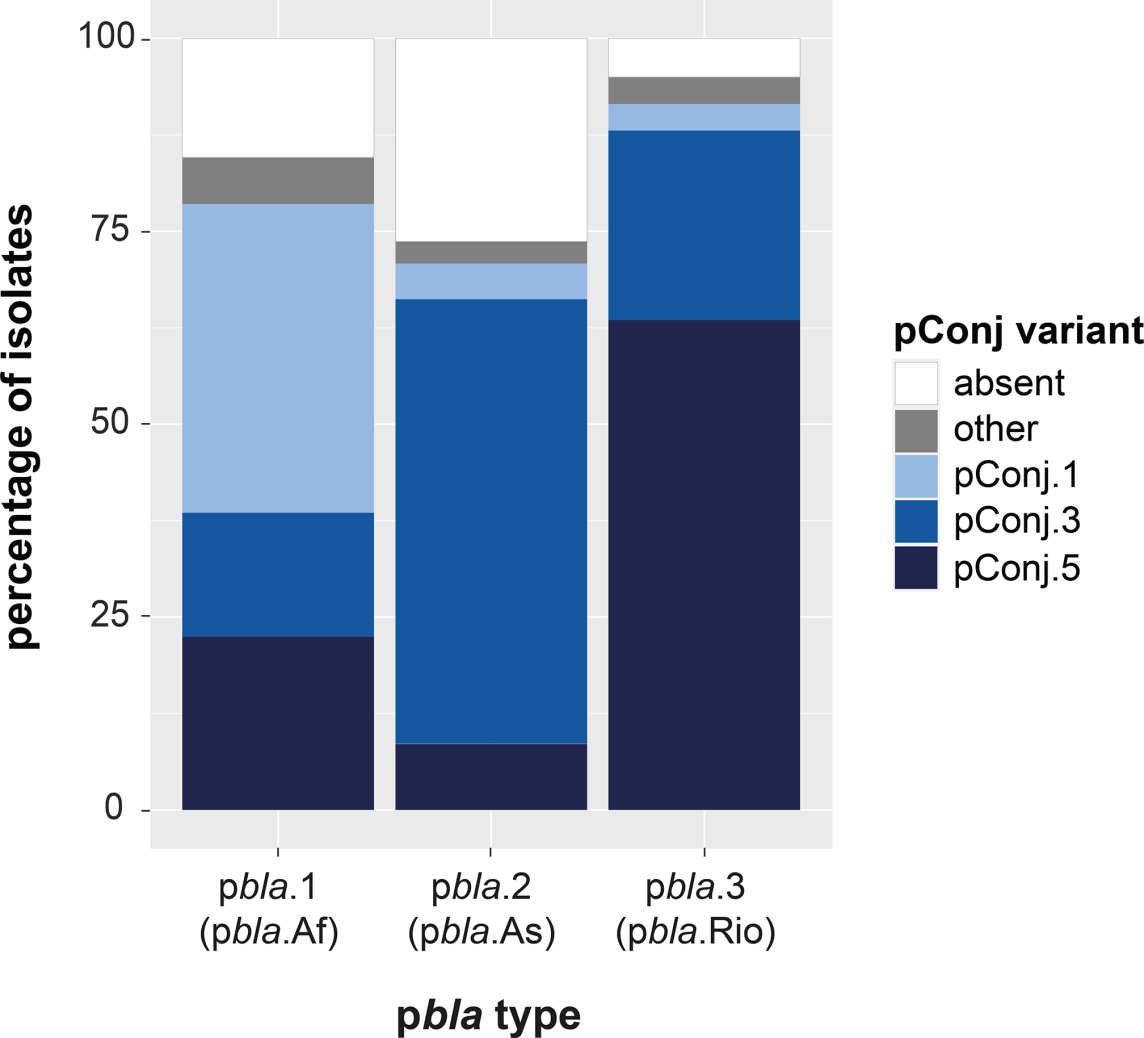
Percentage of p*bla*-carrying isolates with specific pConj variants. p*bla*.1 is found together with pConj variants 1, 3 and 5, while p*bla*.2 is mainly found with variant 3. p*bla*.3 is present in pConj.5 and pConj.3 carrying isolates.

As we described previously [6], the presence of p*bla* is significantly associated with pConj (OR=40.4, *P*<0.001), which has been categorised into seven variants [6]. While pConj variants 1 and 2 differ from 3 and 4 in the *tetM* allele they carry, variants 5, 6 and 7 lack *tetM*, and are referred to as markerless [6]. Furthermore, pConj variants diverge in their toxin-antitoxin systems and parts of the conjugation apparatus. pConj variants 1, 3, 5 and 6 are found across the gonococcal population. In contrast, pConj.2, pConj.4 and pConj.7 are associated with distinct Ng_cgc_400 s [6].

p*bla* is mostly found with pConj.1, pConj.5 and pConj.3, with 30.0, 29.4 and 24.0 % of isolates with p*bla* containing these pConj variants, respectively. Of note, p*bla*.2 is associated with pConj.3 (OR=12.9, *P*<0.001). In contrast, p*bla*.3 is present with pConj.5 (63.5 %) or pConj.3 (22.7 %), while p*bla*.1 is found together with pConj.1, pConj.3 and pConj.5 (Fig. 5). Despite the association between p*bla* and pConj, 15.4 % (425/2758) of all p*bla*-carrying isolates do not harbour pConj. Usually, these are isolates from Ng_cgc_400 s with low (< 30 %) p*bla* carriage. However, isolates from Ng_cgc_400 377, which has a high p*bla* prevalence (52.8 %, 140/265), do not carry pConj.

## Discussion

**Fig. 6.**
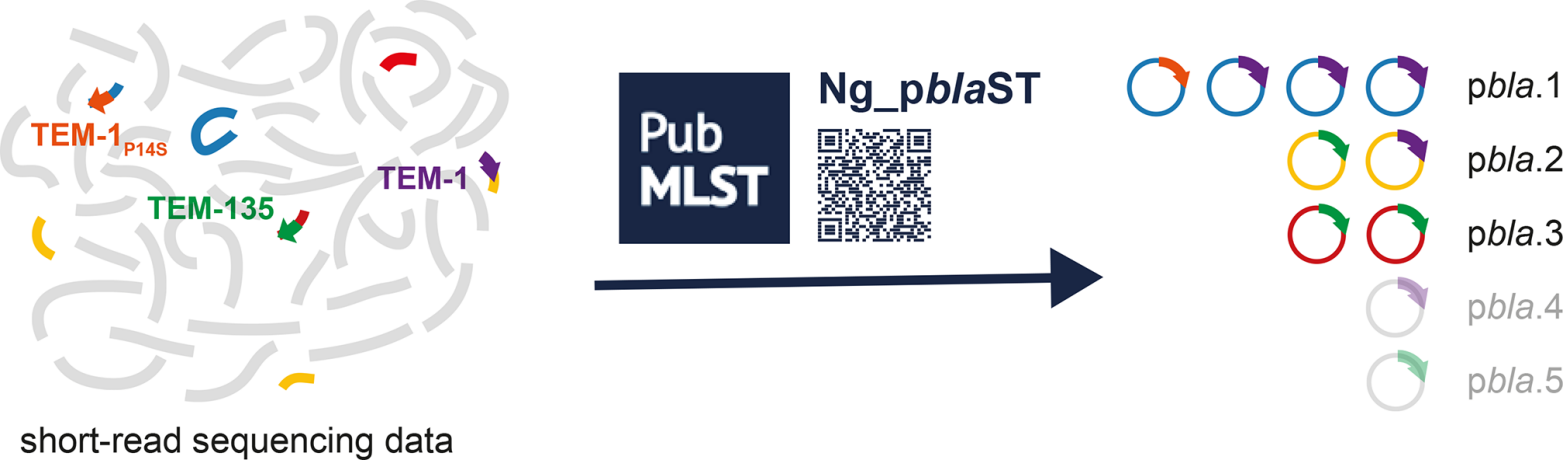
Several variants of the gonococcal β-lactamase plasmid have been described, but little is known about their frequency or distribution in the gonococcal population. We have established and implemented the p*bla* typing scheme Ng_p*bla*ST allowing identification of variants from short read sequencing data. Analysis of 17 827 gonococcal isolates revealed three major variants circulating in gonococcal populations which are associated with distinct TEM alleles.

The potential of the p*bla*-encoded TEM β-lactamase to become an ESBL highlights the importance of monitoring the spread of p*bla* variants in the gonococcal population. However, the presence of repeat regions on the plasmid [14] hinders its assembly from short-read sequences. Additionally, the plasmid sequence is rarely recovered from long-read sequencing data [37], likely due to its small size and low copy number [57]. Therefore, plasmid variants have previously been characterised by their restriction digestion pattern [14] or PCR [20, 21], which only allow small-scale analyses. In this study, we analysed published sequences of p*bla* to design the typing scheme Ng_p*bla*ST to identify plasmid variants from short read WGS according to their characteristic gene presence/absence (Fig. 6). The typing scheme allows plasmid classification from short-read sequencing data and enables unambiguous analysis of large datasets. Current nomenclature of p*bla* variants is based on the geographical region from where they were first identified. However, this scheme is not necessarily informative of their site of origin, and uses geographic names which is no longer recommended [58]. Therefore, we propose a numeric nomenclature for p*bla*, numbering them in the order in which they were first described. We have implemented Ng_p*bla*ST on the PubMLST database and have included both current and proposed nomenclatures to avoid confusion and allow comparison.

We found p*bla*-related sequences that are annotated to be on the chromosome of seven *H. ducreyi* strains. This contrasts with the previous hypothesis that p*bla* was originally a small cryptic plasmid in from *Haemophilus* spp. which gained TEM the β-lactamase through insertion of Tn*2* [59]. Of note, Tn*2* is intact in the p*bla*-related sequences in *H. ducreyi *, indicating that these sequences pre-dated *N. gonorrhoeae* p*bla* in which Tn*2* is truncated. *N. gonorrhoeae* is naturally competent [60] so uptake of linear *H. ducreyi* chromosomal DNA followed by recombination events at repeat sequences could have led to p*bla* appearing in *N. gonorrhoeae *; alternatively, p*bla* was already present as a plasmid in *H. ducreyi* as proposed previously [59].

We analysed the occurrence of p*bla* variants across 15 532 isolates and found that, of the seven described variants, only p*bla*.1, p*bla*.2 and p*bla*.3 are present in appreciable numbers in the gonococcal population. The other plasmid variants were found in 12 isolates only and may either be unstable [18] or are experimental artefacts [19]. Our dataset is the largest quality-checked collection of publicly available isolates, and includes strains from over 60 countries, spanning almost 100 years. However, LMICs, especially those in Africa [22], are under-represented in our and all current datasets. Nevertheless, our dataset does include 317 isolates from ten African countries, expanded by the available WGS data of 49 novel Kenyan isolates. Consistent with previous findings [6], p*bla* prevalence was highest in LMICs. For example, 58.3 % of isolates from countries in Africa harbour the β-lactamase plasmid, compared with only 7.9 % of isolates from the USA and 20 % of isolates from Europe. This was hypothesised to be a result of the extensive spread and maintenance of resistance plasmids in isolates in low-income countries [6]. However, we found that prevalent Ng_cgc_400 s in Africa, such as Ng_cgc_400 21, also have high plasmid carriage when isolated in countries in other geographical regions such as Europe (77.2 %). Therefore, plasmid carriage is an Ng_cgc_400-specific trait and independent of the country from where a strain was isolated. This indicates that p*bla* forms long-term, stable relationships with certain lineages, which may differ in their ability either to acquire and/or maintain the plasmid. Several other factors might contribute to this phenomenon. Compensatory mutations could occur in certain lineages that reduce the metabolic burden imposed by the plasmid [61, 62], while sporadic selection pressure imposed by penicillin, a widely used antibiotic, may select for p*bla* in certain lineages [63]. In contrast, low transfer rates could lead to the absence or loss of the plasmid from other lineages [64]. The preponderance of certain p*bla* variants in geographic regions could result from fitness costs or a selective advantage in specific environmental conditions, leading to the establishment of variants in different areas.

Our analysis also reveals that p*bla* variants co-occur with specific pConj types. Of note, p*bla* is rarely found with pConj variants 2, 4, 6 and 7. These pConj variants are present at lower frequencies than the pConj variants that co-occur with p*bla*, and pConj.2, 4 and 7 are Ng_cgc_400-specific [65]. Therefore, the co-occurrence between p*bla* and pConj variants could merely reflect the wider distribution of pConj variants 1, 3, and 5. Alternatively, specific interactions between p*bla* and pConj variants could explain their co-occurrence. For example, p*bla* MobA has a relaxase domain which nicks the *oriT* sequence on p*bla* [11], but then must interact with the pConj-encoded Type 4 Secretion System for its transfer [66]. Therefore, differences in the *mpf* region of pConj variants [6] may lead to distinct interactions with p*bla* and favour their co-transfer.

p*bla*.3 is associated with TEM-135 and confined to three Ng_cgc_400 s that cluster together on a minimal-spanning tree (Fig. 4). The deletion of the *mob* region indicates this variant might not be mobilised by pConj, with the observed distribution resulting from clonal expansion of the Ng_cgc_400. p*bla*.3 transfer has been reported [67], and co-integration into pConj, mediated by IS*1*, was described as a transfer mechanism of p*bla*.3 in *E. coli* [53, 68]. However, IS*1* is not found in the gonococcal genome, and further experiments are required to investigate possible mechanisms for p*bla*.3 transfer. Nevertheless, p*bla*.3 is found in gonococcal isolates at comparable numbers to p*bla*.2. This might reflect the success of Ng_cgc_400 25, the Ng_cgc_400 with the third most p*bla*-containing isolates in HIC after Ng_cgc_400 21 and 33.

TEM-135 is the second most prevalent allele on p*bla* (in 24.3 % of plasmids) and relative to TEM-1, carries an M182T substitution, which stabilises the enzyme [69] and facilitates further amino acid changes in the active site that would convert the enzyme into an ESBL. We found TEM-135 at almost twice the frequency described before in gonococcal p*bla* [6]. Previous reports indicate an association of p*bla*.3 with TEM-135 [17], while TEM-135 carrying p*bla*.2 has been isolated on a few occasions [57, 70]. Our results confirm these observations but show that almost half of p*bla*.2 carry TEM-135. In contrast, p*bla*.1 encodes mainly TEM-1, with TEM-1_P14S_ found in a subpopulation of p*bla*.1 associated with Ng_cgc_400 33, a prevalent Ng_cgc_400 in HICs. Nothing is known about the effect of this substitution on TEM activity, even though it is common among isolates with p*bla*.1.

As p*bla* long-read sequence data is sparse, we manually validated the typing scheme with p*bla*-containing WHO reference strains, as well as aligning p*bla* contigs to the reference plasmid. This confirmed the typing scheme in every case, albeit with a limited number of isolates. Ng_p*bla*ST is now available on PubMLST, allowing further independent validation by the research community. The typing scheme identifies the five reported deletion variants but should also classify novel variants as untypeable; these should be investigated by PCR or long read sequencing to monitor for the emergence of novel variants which are not included in the current scheme.

In summary, we devised and implemented the typing scheme Ng_p*bla*ST for gonococcal p*bla* and revealed associations of plasmid variants with TEM alleles, pConj variants and Ng_cgc_400 s. Ng_p*bla*ST is available on PubMLST and should aid in monitoring of the spread of the plasmid and TEM alleles, resolve ambiguities [22], and inform research on the adaption of AMR plasmids to their gonococcal host. The high prevalence of plasmid-mediated resistance in LMICs and the potential of p*bla*-encoded TEM to become an ESBL highlights the importance of understanding the variation and distribution of p*bla*. Further research is needed to investigate the interactions of p*bla* with the host genome and co-resident plasmids to understand the underlying factors of its distribution and spread, and to develop interventions to interrupt the emergence of plasmid-mediated β-lactam resistance in this priority pathogen.

## Supplementary Data

Supplementary material 1

Supplementary material 2
