# Supplementary material for: There are three major Neisseria gonorrhoeae β-lactamase plasmid variants which are associated with specific lineages and carry distinct TEM alleles
Source: Microb Genom. 2023 Jul 12;9(7):mgen001057. doi: 10.1099/mgen.0.001057 (PMC10438826; doi:10.1099/mgen.0.001057)
Supplement: Supplementary material 1 [file mgen-9-1057-s001.pdf]

Supplementary Material

Supplementary Figure 1

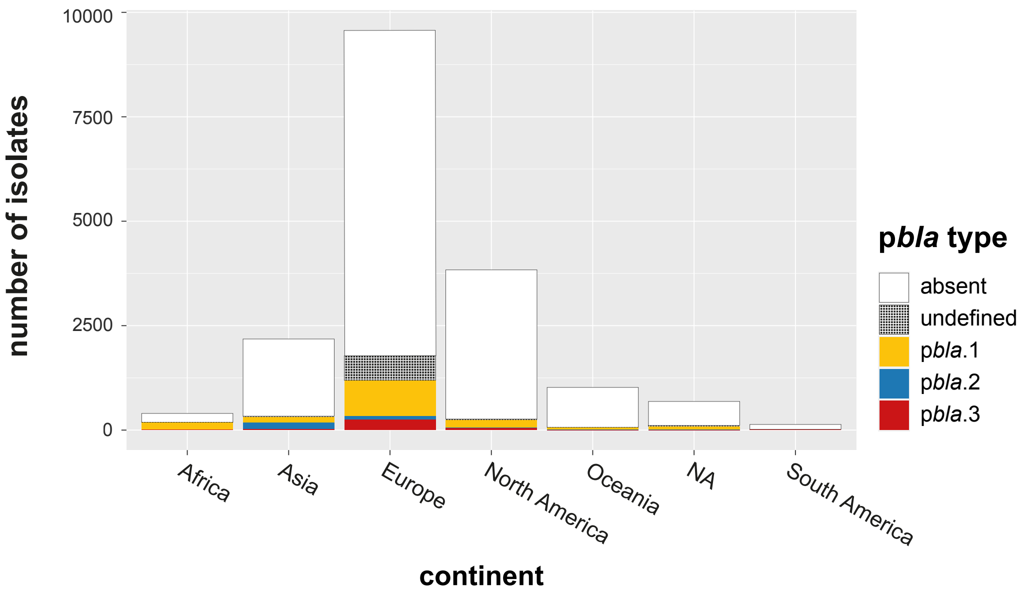

Number of isolates by continent with p*bla* variant carriage indicated in colour. Isolates lacking geographic metadata were classified as NA (not available.

Supplementary Figure 2

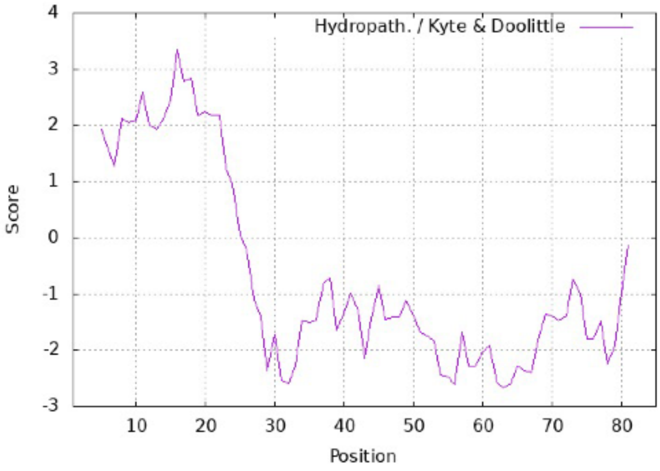

Hydrophobicity plot of NEIS2964 gene product indicates a potential N-terminal transmembrane domain.

Supplementary Figure 3

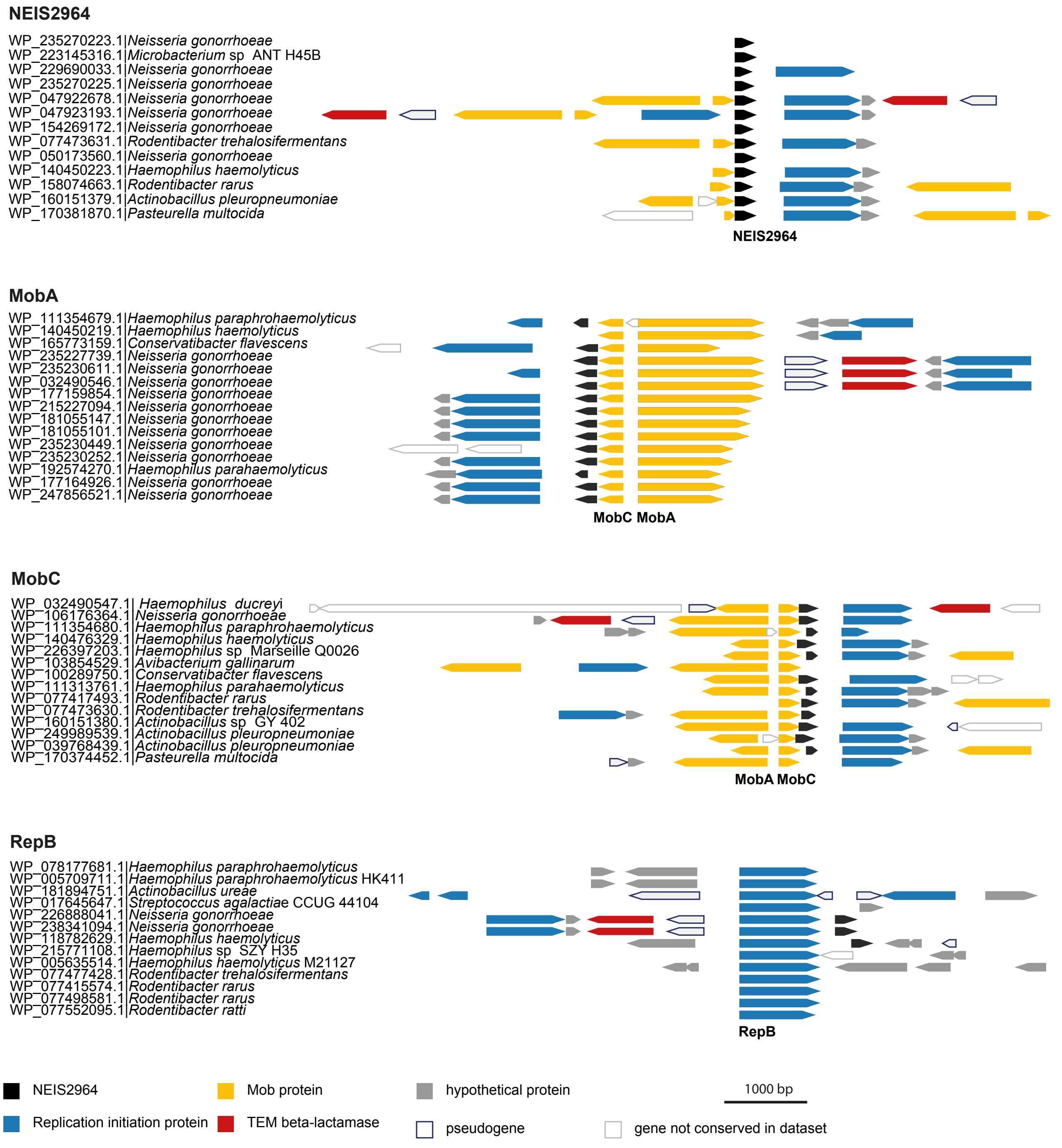

Neighbourhood analysis of NEIS2964, mobA, mobC, repB. WebFlaGS queries the protein sequence against the RefSeq database. The figure shows conserved flanking genes with accession number of sequences homologous to query sequence and species indicated.

Supplementary Table 1

Number of isolates	15,532
Number of Ng_cgcs	230
Number of countries isolates are from	66
Number of isolates with p*bla* (NEIS2960)	2758
Number of isolates with pConj (NEIS2220)	4843
Time covered by isolates	1928-2022 (median = 2014)

Supplementary Table 2

Accessible via figshare: Elsener, Tabea (2023). Supplementary Table 2. figshare. Dataset. https://doi.org/10.6084/m9.figshare.23638992.v1.

Supplementary Table 3

id	isolate	p*bla* type	Ng_cgc	country	year
27417	GCGS0183	p*bla*.3	3	USA	2010
31464	590_10	p*bla*.3	298	Greece	2008
31498	EST_11_14	p*bla*.1	21	Estonia	2001
31527	989000076	p*bla*.3	298	Malaysia	1998
31528	989000021	p*bla*.1	22	Philippines	1998
31550	BX4109	p*bla*.1	21	Pakistan	2010
31568	259000214	p*bla*.1	33	UK	1986
45038	EXNG238	p*bla*.1	21	Australia	2012
48229	SRR3350166	p*bla*.3	25	UK	2015
48263	SRR3350200	p*bla*.3	NA	UK	2014
48265	SRR3350203	p*bla*.3	25	UK	2014
48781	SRR3360809	p*bla*.5	25	UK	2013
106834	ERR3577495	p*bla*.4	17	UK	2015
107199	ERR3577886	p*bla*.4	314	UK	2013
107745	ERR3579047	p*bla*.4	296	UK	2014
115814	14ARS_VSM0248	p*bla*.5	NA	Philippines	2014
